# The surgical management of osteoid osteoma: A systematic review

**DOI:** 10.3389/fonc.2022.935640

**Published:** 2022-07-22

**Authors:** Man Shu, Jin Ke

**Affiliations:** ^1^ Department of Orthopaedics, General Hospital of Southern Theater Command, Southern Medical University, Guangzhou, China; ^2^ Department of Orthopaedics, ZhuJiang Hospital of Southern Medical University, Southern Medical University, Guangzhou, China

**Keywords:** radiofrequency ablation, surgery, cryoablation, microwave ablation, meta-analysis, osteoid osteoma (OO)

## Abstract

**Background:**

Osteoid osteoma (OO) comprises approximately 11%-14% of benign bone tumors. The main symptom of OO is localized pain accompanied by nighttime aggravation. Surgical treatment is frequently used in clinic, including open surgery and percutaneous ablation, the latter including radiofrequency ablation, cryoablation, and microwave ablation, but there is no consensus on when and how to choose the best treatment for OO.

**Purpose:**

We did a systematic review of the literature on existing surgical treatments of OO to assess the safety and efficacy of surgical treatments of OO and to evaluate the surgical options for different locations of OO.

**Methods:**

The inclusion criteria in the literature are 1. Patients diagnosed with osteoid osteoma and treated surgically; 2. Include at least five patients; 3. Perioperative visual analogue scale (VAS), postoperative complications, and recurrence were recorded; 4. Literature available in PubMed from January 2014 to December 2021.

**Results:**

In the cohort, 1565 patients (mainly adolescents) with OO received 1615 treatments. And there are 70 patients with postoperative recurrence and 93 patients with postoperative complications (minor: major=84:9). The results of Kruskal-Wallis examination of each experimental index in this experiment were clinical success rate H=14.818, p=0.002, postoperative short-term VAS score H=212.858, p<0.001, postoperative long-term VAS score H=122.290, p<0.001, complication rate H=102.799, p<0.001, recurrence rate H=17.655, p<0.001, the technical success rate was H=45.708, p<0.001, according to the test criteria of α=0.05, H_0_ was rejected. The overall means of the outcome index in each group were not completely equal.

**Conclusion:**

Percutaneous ablation and open surgery are safe and reliable for OOs, and the technical success rate of percutaneous ablation is higher than that of open surgery. Open surgery and cryoablation can be selected for OOs close to the nerve and atypical sites, while radiofrequency ablation and microwave ablation can be selected for OOs in most other sites.

## Introduction

Jaffe first described osteoid osteoma (OO) in 1935 as a benign isolated osteogenic tumor (1). It accounts for 11%-14% of benign bone tumors (2). OO is most common in the femurs and tibias of adolescents, with 6% spinal lesions (3–5). The main symptom of OO is localized pain that worsens at night. The reason for this is that OO produces a lot of prostaglandin (PG), and PGE increases pain sensitivity (6–9). It recovers on its own, but it takes a long time (10, 11).

Medicines and surgery are used in the medical treatment of OO. The medications used are mostly non-steroidal anti-inflammatory drugs (NSAIDS), which not only provide symptomatic relief but also shorten the time it takes for the body to heal itself (4, 12, 13). On the other hand, long-term use of NSAIDs causes side effects such as bleeding, gastrointestinal reactions, and nephrotoxicity (4).

Open surgery and percutaneous ablation are two surgical options for treating OO. Nonetheless, percutaneous ablation is becoming more popular in hospitals; it is not a replacement for open surgery (14). However, in open surgery, the inexact location and the large surgical incision cause several bone defects that may require bone grafting or internal fixation, increasing the discomfort and expense of the patient (4, 15).

In 1992, D. Rosenthal described the use of radiofrequency ablation (RFA) (16), and since then, percutaneous ablation has become the ‘gold standard treatment’ for OO (17–20). RFA causes tumor cell necrosis due to resistive electrothermal effects and has been shown in clinical trials to be a safe, efficient, and low-cost treatment for OO (18, 21). For the first time, in 2010, the cryoablation was presented to treat OO, which involved freezing and thawing cycles to kill tumor cells (22). This therapy can be ablated in the eccentric position of the lesion, avoiding bone drilling (23, 24), removing the risk of permanent nerve damage, and eventually improving the safety of atypical OO sites (24, 25). Microwave ablation (MWA), another treatment method for OO, was first reported in 2014. Microwave needles emit magnetic fields that generate heat, causing tumor cell necrosis through vibrations generated in surrounding polar molecules (20, 26). MWA has several advantages over RFA, including a faster heating rate, a higher intratumor temperature, a larger ablation range, little effect on tissue, and carbonization (20, 27, 28).

There is no agreement on when and how to select the best treatment for OO. Therefore, this study aims to assess the safety and efficacy of OO surgical treatments. A systematic review of the existing literature on surgical treatments for OO was also used to evaluate the surgical options for different locations of OO.

## Materials and methods

### Selection of studies

The inclusion criteria in the literature are 1. Patients diagnosed with OO and treated surgically; 2. Include at least five patients; 3. Preoperative and postoperative visual analogue scale (VAS), postoperative complications, and recurrence were recorded; 4. Literature available in PubMed from January 2014 to December 2021. Exclusion criteria: 1. Includes ambiguous clinical data. 2. Patients misdiagnosed as OO. 3. Systematic reviews and meta-analysis.

Since the PubMed database described the first case of treating OO by MWA in 2014, we searched the literature published from January 2014 to December 2021. A search algorithm was developed based on a combination of keywords (‘osteoid osteoma’ [All Fields] AND (‘cryoablation’ [All Fields] OR ‘radiofrequency’ [All Fields] OR ‘microwave’ [All Fields] OR ‘surgery *’ [All Fields] OR ‘resection’ [All Fields]) AND (2014: 2021 [update]).

**Figure 1:**
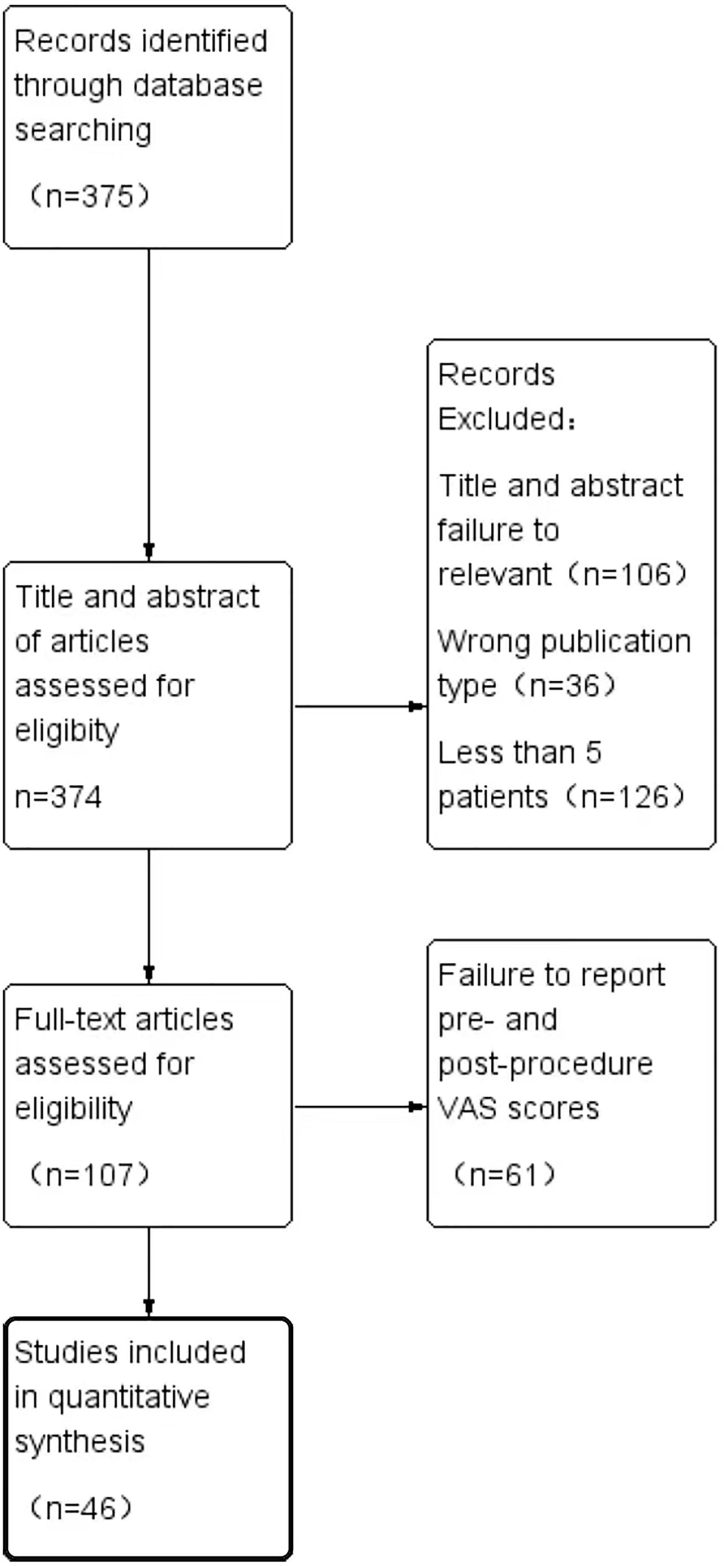
PRISMA flow diagram demonstrating the selection process of articles.

Two authors reviewed the literature (Man Shu and Jin Ke). First, the titles and abstracts of the literature were divided and organized. Furthermore, their full texts were filtered using the aforementioned criteria. The data were extracted by two authors (Man Shu and Jin Ke), and any content disagreements were resolved by a third author. The screening steps are depicted in **Figure 1** of the PRISMA flow diagram. We collected a few parameters as a whole data set, including the total number of patients, patient age and sex, treatment methods, clinical success rate (mean [SD]), changes in perioperative VAS (mean [SD]), complications, and recurrence during follow-up.

### Data analysis

Technical success is defined as ‘cases without any technical failure, such as failure of the range to penetrate the nidus, machine failure during surgery, etc.’, while clinical success is defined as ‘resolution of the patient’s symptoms throughout the follow-up period’. The recurrence rate is the percentage of cases that relapse. The total number of technical successes is divided by the total number of cases reported by each study to calculate the technical success rate. The total number of clinical successes is divided by the total number of cases reported by each study to get the clinical success rate. The ‘short-term postoperative VAS’ is defined as the most recent postoperative VAS, while the ‘long-term postoperative VAS’ is defined as the last postoperative follow-up VAS. The second treatment after treatment failure was counted as one patient and two surgeries, and if the second other treatment was received, in each of the two treatment modalities, there was one patient and one operation in each method.

The primary endpoints for this study were postoperative VAS scores and clinical success rate, with complications and recurrences as secondary endpoints. We compared VAS scores and clinical success rates between groups to assess the efficacy of each surgical method. The rate of complications was calculated after complications were classified using the Society of Interventional Radiology (SIR) classification system for complications (29). The mean and standard deviations (SD) of perioperative VAS and clinical success rates were calculated, and data for each patient were recorded separately if they were not reported in this study. We used SPSS 25.0 for Kruskal-Wallis testing and the Kruskal-Wallis one-way ANOVA method for postmortem multiple comparisons to assess differences between groups.

## Results

### Study selection

Approximately 375 articles were chosen from the PubMed database. According to the abstract screening, 106 articles were not related to the purpose of the current study, 36 articles belonged to a review, and 126 articles had fewer than five patients. The full texts of the remaining 107 articles were reviewed, excluding the 61 articles that did not include a perioperative VAS score. The PRISMA flow diagram depicts the process of screening for inclusion (**Figure 1**).

### Patient population

**Figure 2:**
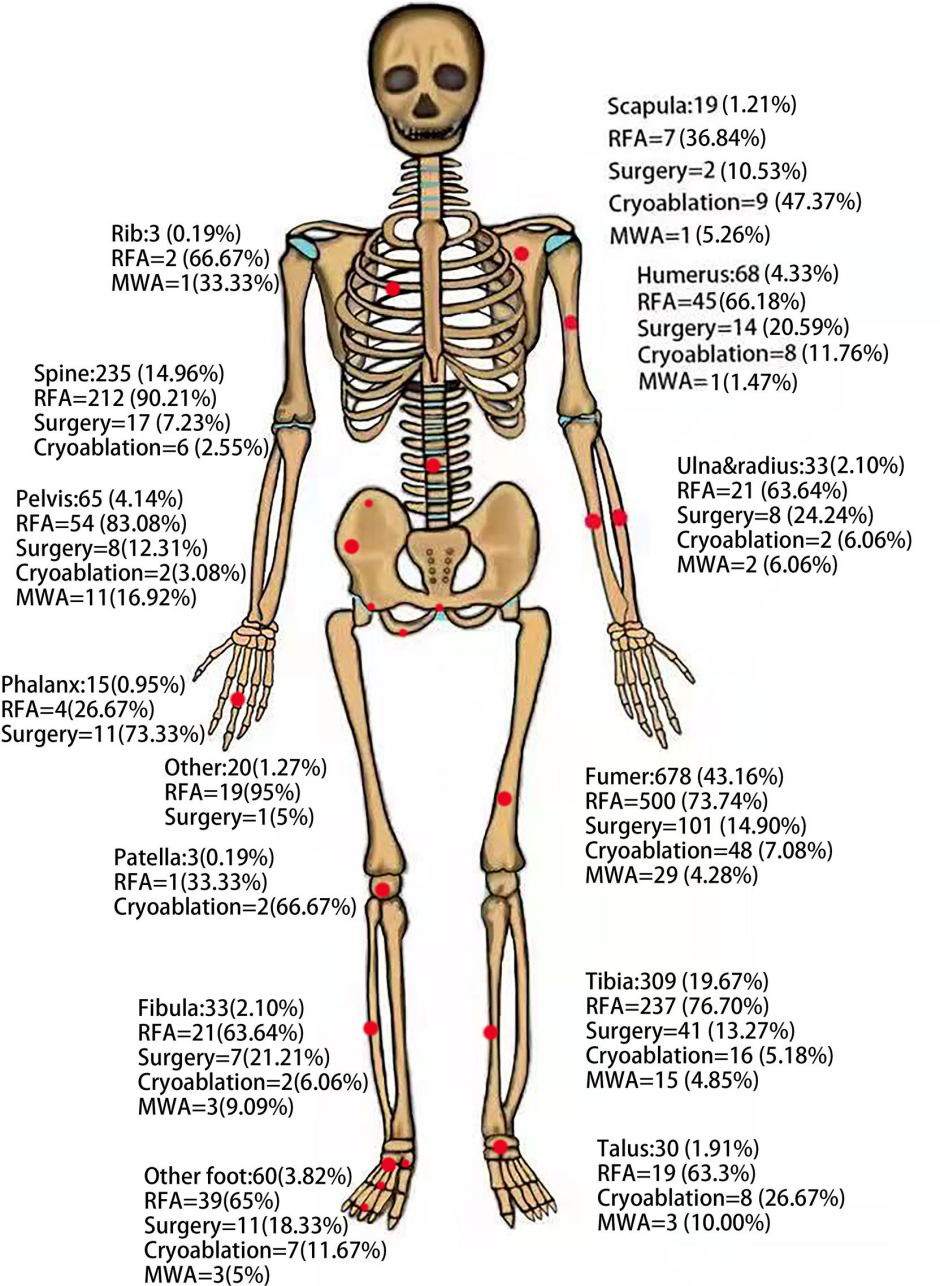
Anatomic distribution of osteoid osteomas in the patient cohort based on technology.

A total of 1615 treatments were administered to 1565 patients with OO. The included patients ranged in age from 3 to 68 years, with the majority being adolescents. **Figure 2** shows the anatomical distribution of OO. **Table 1** lists the outcome indicators for each study. Individual OO of the spine (RFA: surgery = 7:2, population ratio was 185:30) was recorded in nine of the included studies. OO of atypical sites was performed separately in three studies (RFA: surgery = 2:1, population ratio was 89:26), and four studies included pediatric patients (RFA: surgery: cryoablation = 2:1:1, population ratio was 40:47:29).

**Table 1:** Characteristics of the results of each study.

Study	Reference no.	Mean follow-up time (m)	Mean lesion size (mm)	VAS pre-procedure	VAS recent post-procedure	VAS last post-procedure	Clinical success	Complication rate	Recurrence rate
Basile, A	(26)	8.7	7.3	6	0	0.3	100.0%	0.0%	0.0%
Coupal, T. M	(23)	6	9.9	7.4	1.5	0.3	100.0%	0.0%	0.0%
Morassi, L. G	(30)	23.2	NA	8.6	1	0	86.7%	0.0%	15.4%
Regev, G. J	(31)	18	14	7.7	2.8	0	100.0%	0.0%	0.0%
Yu, F	(32)	15.5	NA	3.4	0.8	0.1	100.0%	0.0%	0.0%
Alemdar, C	(33)	53.5	NA	8.1	0.8	1.6	77.4%	7.6%	9.4%
Arıkan, Y	(34)	15.8	6.9	7.2	0.64	0.64	82.4%	11.8%	17.7%
Filippiadis, D	(35)	6	9.1	8.9	0	0	100.0%	0.0%	0.0%
Gökalp, M. A	(36)	12	NA	8.3	0.5	0	100.0%	0.0%	0.0%
Guo, X	(37)	20	NA	6.5	1.5	0	100.0%	0.0%	0.0%
Karagöz, E	(38)	26.5	8.1	9	0	0	94.4%	11.1%	5.6%
Lin, N	(39)	16	1~5	4.7	1.4	0	100.0%	0.0%	0.0%
Masciocchi, C	(40)	24	NA	8.5	1.5	0	100.0%	6.7%	0.0%
Miyazaki, M	(41)	15.1	9.9	7.1	1.6	0.2	86.0%	57.1%	0.0%
Outani, H	(42)	18	9	7.3	0	0	96.8%	9.4%	3.1%
Whitmore, M. J	(43)	18.3	6.7	10	0	0	90.5%	20.7%	3.5%
Albisinni, U	(44)	41.5	11.4	8	0	0	93.4%	3.3%	6.6%
Chahal, A	(45)	15.4	8.5	7	0	0	86.2%	2.3%	13.8%
Costanzo, A	(46)	84.3	10	7.4	0.3	0	100.0%	0.0%	0.0%
Erol, B	(47)	59	NA	7.7	0.3	0	100.0%	0.0%	0.0%
Faddoul, J	(48)	12~84	9.9	7.6	2.56	0	87.5%	0.0%	12.5%
Kulkarni, S. S	(49)	48	NA	7.8	0.4	0	97.7%	7.0%	2.3%
Nöel, M. A	(50)	12	9.9	8.8	2	0	85.7%	0.0%	14.3%
Prudhomme,C	(5)	1	5.7	6.46	0.85	0.46	92.3%	15.4%	7.7%
Wang, B	(51)	46.6	10.3	7.6	0	0.3	100.0%	0.0%	0.0%
Wu, H	(52)	12	8	3.2	4.5	2.2	72.2%	27.8%	8.3%
Hage, A. N	(53)	93.1	9.4	8	0	0	91.3%	2.2%	6.5%
Santiago, E	(54)	21	7.8	8	0	0	95.2%	14.3%	4.8%
Ankory, R	(55)	36	NA	7.7	0.5	0	94.2%	1.9%	5.8%
Beyer, T	(56)	28.5	NA	6.2	0.71	0	89.7%	2.6%	9.1%
Fujiwara, T	(14)	25	NA	7	2.2	0	100.0%	0.0%	0.0%
Kaptan, M. A	(57)	17.8	11.84	8.6	0.1	0	100.0%	25.0%	0.0%
Kostrzewa, M.	(58)	36	5.3	6.9	1.25	0	91.7%	4.2%	4.2%
Neyisci, C	(59)	16	NA	8.3	1.23	0	100.0%	9.5%	0.0%
Sahin, C	(60)	23	7~15	8	0~1	0	98.0%	6.0%	1.7%
Yu, X	(61)	55.5	11.3/13	8/6.5	1/2	0.75/0	100%/93.8%	0.0%/18.8%	0.0%/6.3%
Ayas, M. S	(62)	12	NA	4.8	0.2	0.2	100.0%	18.8%	6.3%
Reis, J.	(63)	12	10/11	7/8	0/0.2	0.4/0.8	93.3%/93.3%	13.3%/0.0%	6.7%/6.7%
Tanrıverdi, B	(64)	46	NA	7.2	1.3	0	100.0%	0.0%	0.0%
Yuce, G	(65)	22	3.6	8.4	3.2	3.2	96.4%	1.8%	3.6%
Arrigoni, F	(66)	26	NA	9.1	0	0	98.4%	1.6%	1.6%
Filippiadis, D	(67)	23.3	8.28	9.1	0	0	100.0%	0.0%	0.0%
Le Corroller, T	(24)	18~90	6	8	0	0	96.0%	6.0%	4.0%
Lorenc, T	(68)	90	5.6	8.5	0	0	87.5%	7.7%	15.4%
Niazi, G. E	(69)	24	6.1	8.6	0	0	100.0%	2.9%	0.0%
Somma, F	(70)	24	NA	8.3	1.5	0.47	96.1%	5.9%	3.9%

### Outcomes

**Table 2** describes the characteristics of the patients and each endpoint. **Table 3** shows the total clinical success rate in studies that recorded atypical sites alone [excluding femur and tibia (64, 69)] of OO. This study included 70 patients with postoperative recurrence and 93 patients with postoperative complications (minor: major=84:9).

**Table 2:** Patient characteristics and outcomes.

	RFA	Surgery	Cryoablation	MWA	Total
Patients (n)	1161	235	110	59	1565
Male : female	804 : 357	161 : 74	69 : 41	37 : 22	1071:494
Age (mean ± SD)	20.6 ± 4.6	17.1 ± 6.1	22.1 ± 6.1	22.8 ± 4.5	20.3 ± 5.2
lesion size (mm)	9.0 ± 2.2	9.2 ± 3.8	6.9 ± 1.2	6.8 ± 2.0	8.6 ± 2.4
VAS scores					
Preoperative	7.8 ± 1.1	6.5 ± 1.8	8.5 ± 0.9	6.7 ± 0.3	7.6 ± 1.3
postoperative short-term	0.7 ± 0.8	1.5 ± 1.5	1.4 ± 0.4	0.7 ± 0.6	0.8 ± 1.0
postoperative long-term	0.2 ± 0.7	0.7 ± 0.9	0.3 ± 0.1	0.2 ± 0.2	0.3 ± 0.7
Clinical success (95%CI)	94.8% (94.5%, 95.1%)	90.1% (88.6%,91.7%)	94.9% (94.4%,95.4%)	93.3% (92.6%,93.9%)	94.0% (93.7%,94.3%)
Recurrences (95%CI)	4.8% (4.5%, 5.1%)	3.7% (3.1%, 4.2%)	3.6% (3.3%, 3.8%)	5.1% (4.5%, 5.8%)	4.5% (4.3%,4.8%)
Technical success (95%CI)	98.1% (97.9%, 98.3%)	95.8% (95.1, 96.6%)	99.1% (98.9%,99.3%)	100% (100%,100%)	97.9% (97.7%, 98.1%)
Complications (95%CI) Follow-up (mean ± SD) Biopsy	5.1% (4.7%, 5.6%) 32.4 ± 22.4 68.6% (393/573)	7.4% (6.1%, 8.7%) 35.1 ± 21.0 82.9% (165/199)	10.9% (9.6%, 12.3%) 33.9 ± 18.8 52.8% (19/36)	8.3% (6.8%, 9.8%) 18.9 ± 14.8 72.9% (10/13)	6.0% (5.6%, 6.4%) 32.4 ± 21.8 71.5% (587/821)

**Table 3:** The clinical success rate of OO in the atypical sites.

	Surgical resection	RFA	Total
clinical success rate of OO in the spine	96.7% (95.5%, 97.9%)	91.5% (91.0%, 92.1%)	92.2% (91.7%, 92.8%)
clinical success rate of atypical sites	100%	97.8% (97.4%, 98.2%)	98.3% (97.9%, 98.6%)

Among the 54 patients who relapsed after RFA, 43 were cured after secondary RFA, nine with open surgery, one with MWA, and one with laser ablation. Nine patients relapsed after open surgery, three were cured by secondary surgery, one by RFA, and five were not recorded. One of the four patients who relapsed after cryoablation was cured with RFA, while the other three were cured with secondary cryoablation. While three patients relapsed after MWA, one underwent surgical resection, one was cured by secondary MWA, and one was not recorded. The overall rate of recurrence in 12 cases of atypical OO (including spine) was 5.5% (n = 18), of which the rate of recurrence after RFA was 6.2% (n = 17), six were cured by RFA again after relapse, three were cured by open surgery, and others were not recorded; the rate of recurrence after open surgery was 1.8% (n = 1), and one case was cured with RFA 2 years later.

The SIR system was used to classify complications. Among postoperative complications of RFA (minor: major=51:8), 21 were grade A (five transient pain and paresthesia, one muscle hematoma, one soft-tissue edema, one skin erythema, one needle tip rupture, 12 abnormalities of the transient blood biochemical index), 29 were grade B (21 burns, six infection, one fasciitis, one herniated lumbar disc herniation), and eight were grade D (three of osteomyelitis, two fractures, one thigh abscess, one pulmonary edema, one peroneal nerve injury). Postoperative complications of open surgery (minor: major =16:1), four of grade A (four of temporary dysfunction), 12 of grade B (six of infection, three of neurovascular injury, two of limited activity induced by pain, and one of delayed healing), and one of grade D (fracture). Among the post-cryoablation complications (minor = 12), four were of grade A (transient pain and soft tissue swelling), two were of grade B (mild burns), and the data of six were not recorded in detail. All postoperative complications of MWA (minor = 5) were grade A (two paresthesia, two mild burns, and one hypofunction).

The Kruskal-Wallis test results for each outcome in this experiment are provided below. According to the test criteria of α= 0.05, the clinical success rate was H=14.818, p=0.002, the postoperative short-term VAS score was H=212.858, p<0.001, the postoperative long-term VAS score was H=122.290, p<0.001, rate of complication was H=102.799, p<0.001, rate of recurrence was H=17.655, p=0.001, the technical success rate was H=45.708, p<0.001, H_0_ was rejected, and it can be considered that the overall mean of each outcome index in each group was not completely equal. **Table 4** shows pairwise comparisons of the outcome measures in each group.

**Table 4:** Results of pairwise comparison of outcome measures in each group.

	RFA-Cryoablation	RFA-MWA	RFA-surgery	MWA-Cryoablation	MWA-surgery	Cryoablation-surgery
Clinical success rates	H=78.30 P=0.471	H=201.44 P=0.004	H=-15.91 P=1.000	H=-123.14 P=0.523	H=-217.35 P=0.005	H=94.212 P=0.405
Recurrence rate	H=28.72 P=1.000	H=-139.23 P=0.114	H=103.03 P=0.007	H=167.95 P=0.116	H=242.26 P=0.001	H=-74.31 P=0.889
Complication rates	H=-384.50 P<0.001	H=-294.89 P<0.001	H=47.65 P=0.810	H=-89.602 P=1.000	H=342.55 P<0.001	H=-432.15 P<0.001
Postoperative short-term VAS	H=415.06 P<0.001	H=-63.59 P=1.000	H=-316.59 P<0.001	H=478.65 P<0.001	H=-252.97 P=0.001	H=731.62 P<0.001
Postoperative long-term VAS	H=116.28 P=0.006	H=-229.84 P<0.001	H=-231.86 P<0.001	H=346.13 P<0.001	H=-2.02 P=0.001	H=348.14 P<0.001
Technical success rate	H=40.62 P=1.000	H=-229.38 P<0.001	H=115.94 P<0.001	H=270.00 P<0.001	H=345.32 P<0.001	H=-75.32 P=0.465

The P-value in the table is adjusted.

### Ablation process and follow-up

**Table 5** shows the operating and hospitalization times of the patients in each group. The average intraoperative control temperature of 826 patients in 24 studies of RFA treatment was 90°C and continuously heated for 6.7 ± 3.3 min. A freezing-thawing cycle was used to treat the 100 patients with cryoablation. The average freeze time was 10 min, and the average thaw time was 7.3 min. In the three MWA studies, the power of 16W, 80°C ablation was used for 76 ± 53.26s; 20W, 80°C ablations for 2 min; and 50W ablation for 1 min or 60W ablation for 1.7 min.

**Table 5:** Mean length of surgery and hospital stay.

		Patients	Mean	SD	SEM
operation time(minutes)	RFA	358	72.7	20.19	1.07
	Surgery	149	70.1	45.26	3.71
	Cryoablation	71	80.0	0.00	0.00
	Total	578	72.9	28.03	1.17
length of stay(days)	RFA	548	1.3	2.02	0.09
	Surgery	166	2.1	1.36	0.11
	Cryoablation	50	0.4	0.50	0.07
	Total	764	1.4	1.87	0.07

RFA, MWA, and surgical resection were found, respectively, only in one patient with recurrence after 2 years of follow-up.

## Discussion

In this study, the technical success rate of each surgical method was positively correlated with clinical success. Prud’Homme et al. (5) documented a clinical failure of a patient with OO at the ankle due to slight intraoperative movement; Le Corroller et al. (24) documented two failed cases, one of which was due to the unsatisfactory position of the freezing probe. Chahal et al. (45) documented postoperative recurrence in nine patients with poor localization. The current study found that percutaneous ablation had a higher technical success rate than open surgery. The main reasons for the failure of each technology were positioning issues and puncturing issues. Therefore, it also demonstrates that technological failure is a major cause of clinical failure and recurrence. To improve the effectiveness of surgery, we can choose to perform it under computer tomography (CT) guidance multiple times, and we can combine it with 3D reconstruction to design the puncturing process.

Outani et al. (42) recorded two postoperative fractures and one postoperative osteomyelitis among the major complications in this study. A case of fibula fracture occurred 10 days later as a result of the addition of two additional holes at the ablation site by 3D navigation that increased bone defect; one case was fracture caused by intense exercise 5 weeks after the operation, and one case had osteomyelitis at the ablation site 2 weeks later. Alemdar et al. (33) recorded incomplete fractures caused by exercise within 3 months after the operation. Yuce et al. (65) reported osteomyelitis caused by burn infection caused by needle overheating. Kaptan et al. (57) documented a case of local osteomyelitis without cause. Based on the foregoing, several measures can be implemented to prevent the occurrence of serious complications and thus improve the safety of surgical treatment, such as preoperative iodine coating to prevent postoperative infection (71), reducing bone defects during operation, limiting exercise within 3 months after the operation, using sterile ice packs to cool the surrounding skin during percutaneous ablation or inserting additional needles to infuse saline to protect peripheral nerves (72, 73), or multiple low power ablations.

### Surgical modalities

This study demonstrated that open surgery and percutaneous ablation are safe and reliable procedures (18, 21).

RFA has become the most widely used method for treating OO in recent years and is considered the gold standard. RFA was used to treat approximately 74.2% of the cases in this study. Even for the OO near nerve sites and other atypical sites, the success rate was 91.5% and 97.8%, demonstrating the success of RFA in OO treatment. However, the use of ground pads in RFA increases the risk of skin burns.

The success rate of open surgery for OO adjacent to important neurovascular sites and atypical sites was 96.7% and 100%, respectively. Therefore, open surgery remains a viable option for OO near neurovascular and atypical sites. Open surgery is also constantly evolving: CT-guided drilling resection (33) and CT-guided Kirschner wire positioning (36). Nevertheless, patients suffer more trauma in open surgery.

Compared to RFA, conscious patients tolerated cryoablation well, which can significantly reduce postoperative pain and hospitalization time (54). Cryoablation has the potential to reduce the risk of permanent nerve damage. Le Corroller et al. (24) found no neurological damage following spinal OO cryoablation. Therefore, cryoablation is preferred for OO near atypical sites. The procedure is so time-consuming that it lengthens the duration of the operation and thus increases the likelihood of complications (71).

In this study, 74.6% (44/59) of OO occurred in the MWA group at typical sites (femur and tibia). Budrevicius et al. (74) reported successful MWA treatment in one of the OO cases at the joint site L3 (not included in this study). MWA of OO in atypical sites (including the spine) is theoretically equally effective. MWA had less power than RFA in this study, had a shorter ablation time, and had no infection or serious complications after ablation. Therefore, it is concluded that MWA is a reliable therapy for OO at common sites. However, in this study, the incidence of burns in MWA (3.4%) is higher than that of RFA (1.8%), which may be due to the rapid heating of MWA (75).

### Biopsy and follow-up

Although tumor pathology is usually the gold standard, some doctors insisted that a biopsy was unnecessary due to the typical symptoms and imaging characteristics of OO. However, in the study of Regev et al. (31), one patient with Ewing’s sarcoma was misdiagnosed as OO, and in the Reis et al. (63) study, a patient with suspected OO was pathologically diagnosed with osteosarcoma (this patient was not included in the study). In any case, while a biopsy is not always necessary for the diagnosis of OO, it is significant to rule out other diseases.

OO recurrence is most common within the first 2 years after surgery (76, 77). After 24 months, approximately three of 72 recurrences occurred in this experiment. This reflects the importance of follow-up as well as the reference significance of at least a 24-month follow-up period.

### Limitations

This study has several limitations. First, there was the impact of systematic and random errors on the validity of the study results. Second, the article only included studies with five or more patients from 2014 to 2021, resulting in a limited number of original articles in the literature. Third, fewer cases of cryoablation and MWA for the treatment of OO were reported, limiting the ability to compare different treatment methods.

### Conclusion

In conclusion, open surgery and percutaneous ablation, such as RFA, MWA, and cryoablation, are appropriate and safe. Percutaneous ablation has been found to have a higher technical success rate than open surgery. Open surgery and cryoablation are effective for OO near nerve sites and in atypical sites, whereas RFA and MWA are beneficial for OO in most typical sites.

## Data availability statement

The datasets presented in this study can be found in online repositories. The names of the repository/repositories and accession number(s) can be found in the article/supplementary material.

## Author contributions

JK contributed to conception and design of the study. MS and JK screened and extracted data. MS wrote the first draft of the manuscript. MS and JK contributed to manuscript revision, read, and approved the submitted version.

## Funding

This work was supported by The Natural Science Foundation of Guangdong Province, China (No. 2020A1515010625)

## Conflict of interest

The authors declare that the research was conducted in the absence of any commercial or financial relationships that could be construed as a potential conflict of interest.

## Publisher’s note

All claims expressed in this article are solely those of the authors and do not necessarily represent those of their affiliated organizations, or those of the publisher, the editors and the reviewers. Any product that may be evaluated in this article, or claim that may be made by its manufacturer, is not guaranteed or endorsed by the publisher.
